# Supplementary material for: Long non-coding RNA Neat1 and paraspeckle components are translational regulators in hypoxia
Source: eLife. 2022 Dec 22;11:e69162. doi: 10.7554/eLife.69162 (PMC9799981; doi:10.7554/eLife.69162)
Supplement: Supplementary file 8. — HL-1 cardiomyocytes were transfected with gapmer Neat1, Neat1_2, or control. Polysomes were purified on sucrose gradient as described in Star Methods. RNAs were purified from cytoplasmic extracts and from pooled polysomal fractions and analyzed on a Fluidigm deltagene PCR array from two biologicals replicates (cell culture dishs and cDNAs), each of them measured in three technical replicates (PCR reactions). mRNA levels in polysomes (polysomal RNA/ total RNA) were analyzed for each gene. Relative quantification (RQ) of mRNA level was calculated using the 2–ΔΔCT method with normalization to Gapdh mRNA and to HL-1 tranfected by gapmer control, and is shown as fold change of expression. RQ1 and RQ2 correspond to two independent experiments. To measure the fold change of repression, the mean is expressed as –1/RQ for the values <1 (yellow column). [file elife-69162-supp8.docx]

| \| **A/ *Neat1* knock-down** \| \| \|  \|  \|  \|  \|  \|  \|  \| \| --- \| --- \| --- \| --- \| --- \| --- \| --- \| --- \| --- \| --- \| \|  \| \|  \|  \|  \|  \|  \|  \|  \|  \| \|  \|  \| \|  \|  \|  \|  \|  \|  \|  \| \|  \| **Total mRNA** \| \| \| **Polysome bound mRNA** \| \| **Fold change** \| \| **Fold change Mean** \| \| \| **Gene name** \| RQ 1 \| \| RQ 2 \| RQ 1 \| RQ 2 \| RQ (polysomes)/ RQ (Total mRNA) 1 \| RQ (polysomes)/ RQ (Total mRNA) 2 \| RQ (ou -1/RQ) \| SD \| \| *Aanat* \| ND \| \| 87.95 \| 1.58 \| ND \| ND \| ND \| ND \| ND \| \| *Adar (Adar1)* \| 1.35 \| \| 0.88 \| 1.14 \| 0.99 \| 0.85 \| 1.12 \| -1.02 \| 0.14 \| \| *Alox15* \| ND \| \| 0.22 \| 4.17 \| 0.61 \| ND \| 2.75 \| ND \| ND \| \| *Ang* \| 0.80 \| \| 1.00 \| 0.50 \| 0.82 \| 0.63 \| 0.82 \| -1.38 \| 0.09 \| \| *Apaf1* \| 1.21 \| \| 0.93 \| 1.50 \| 1.17 \| 1.24 \| 1.25 \| 1.25 \| 0.01 \| \| *Apln* \| 1.36 \| \| 0.99 \| 1.07 \| 1.23 \| 0.79 \| 1.24 \| 1.02 \| 0.22 \| \| *Aplnr* \| 1.15 \| \| 0.22 \| 0.78 \| 0.20 \| 0.68 \| 0.92 \| -1.25 \| 0.12 \| \| *Bag1* \| 1.19 \| \| 1.03 \| 0.75 \| 1.12 \| 0.63 \| 1.09 \| -1.16 \| 0.23 \| \| *Bax* \| 1.27 \| \| 1.05 \| 1.00 \| 1.18 \| 0.78 \| 1.12 \| -1.05 \| 0.17 \| \| *Bcl2 (Bcl-2)* \| 1.02 \| \| 0.96 \| 0.56 \| 0.63 \| 0.55 \| 0.65 \| -1.66 \| 0.05 \| \| *Bcl2L1 (Bcl-XL)* \| 1.25 \| \| 1.09 \| 1.42 \| 0.94 \| 1.13 \| 0.86 \| -1.00 \| 0.14 \| \| *Bip(GRP78/Hspa5)* \| 1.18 \| \| 0.64 \| 0.95 \| 0.92 \| 0.80 \| 1.45 \| 1.13 \| 0.33 \| \| *BNP (Nppb)* \| 3.36 \| \| 0.93 \| 1.45 \| 1.88 \| 0.43 \| 2.04 \| 1.23 \| 0.80 \| \| *Casp2 (Caspase-2)* \| 0.95 \| \| 0.42 \| 1.08 \| 0.82 \| 1.14 \| 1.94 \| 1.54 \| 0.40 \| \| *Cdk1* \| 0.97 \| \| 0.72 \| 0.78 \| 0.88 \| 0.80 \| 1.23 \| 1.01 \| 0.21 \| \| *Cdkn1B (p27kip1)* \| 0.87 \| \| 0.86 \| 0.75 \| 0.89 \| 0.87 \| 1.03 \| -1.05 \| 0.08 \| \| *Cdkn2A (p16INK4)* \| 1.08 \| \| 1.01 \| 0.69 \| 0.90 \| 0.64 \| 0.89 \| -1.31 \| 0.13 \| \| *Col1A1* \| 1.06 \| \| 0.69 \| 0.95 \| 0.73 \| 0.90 \| 1.06 \| -1.02 \| 0.08 \| \| *Col3A1* \| 0.80 \| \| 0.54 \| 1.13 \| 0.55 \| 1.41 \| 1.01 \| 1.21 \| 0.20 \| \| *Csde1* \| 1.07 \| \| 0.72 \| 0.84 \| 0.89 \| 0.78 \| 1.23 \| 1.01 \| 0.23 \| \| *Ctgf* \| 1.02 \| \| 0.79 \| 1.05 \| 1.09 \| 1.03 \| 1.39 \| 1.21 \| 0.18 \| \| *Cyr61* \| 1.60 \| \| 1.37 \| 1.78 \| 1.34 \| 1.11 \| 0.97 \| 1.04 \| 0.07 \| \| *Dazap1* \| 0.97 \| \| 1.11 \| 0.71 \| 0.88 \| 0.74 \| 0.79 \| -1.31 \| 0.02 \| \| *Ddx17* \| 1.11 \| \| 0.65 \| 1.15 \| 0.65 \| 1.04 \| 1.00 \| 1.02 \| 0.02 \| \| *Egr1* \| 2.50 \| \| 1.36 \| 1.90 \| 1.08 \| 0.76 \| 0.79 \| -1.29 \| 0.01 \| \| *Eno3* \| 1.12 \| \| 0.78 \| 0.92 \| 0.81 \| 0.83 \| 1.04 \| -1.07 \| 0.10 \| \| *Epas1* \| 1.53 \| \| 0.84 \| 0.89 \| 0.94 \| 0.58 \| 1.12 \| -1.17 \| 0.27 \| \| *Fgf1* \| 0.85 \| \| 0.51 \| 0.81 \| 0.69 \| 0.95 \| 1.36 \| 1.16 \| 0.20 \| \| *Fgf10* \| 1.53 \| \| 0.78 \| 1.12 \| 1.20 \| 0.73 \| 1.53 \| 1.13 \| 0.40 \| \| *Fgf2* \| ND \| \| 1.23 \| 0.19 \| 0.23 \| ND \| 0.19 \| ND \| ND \| \| *Fgf21* \| 1.33 \| \| 0.93 \| 0.49 \| 0.44 \| 0.37 \| 0.47 \| -2.39 \| 0.05 \| \| *Fgf23* \| ND \| \| ND \| 2.38 \| ND \| ND \| ND \| ND \| ND \| \| *Fgf4* \| 0.23 \| \| 193.41 \| 32.32 \| 8.24 \| 139.51 \| 0.04 \| ND \| ND \| \| *Fgfr1* \| 0.94 \| \| 0.51 \| 0.58 \| 0.48 \| 0.62 \| 0.94 \| -1.28 \| 0.16 \| \| *Fgfr2* \| 1.10 \| \| 0.78 \| 0.87 \| 0.67 \| 0.79 \| 0.87 \| -1.21 \| 0.04 \| \| *Fgfr3* \| 1.20 \| \| 0.76 \| 1.07 \| 1.04 \| 0.89 \| 1.38 \| 1.14 \| 0.24 \| \| *Fmr1 (Fmrp)* \| 1.21 \| \| 1.07 \| 0.63 \| 1.02 \| 0.52 \| 0.95 \| -1.36 \| 0.22 \| \| *Fn1 (Fibronectine)* \| 0.88 \| \| 0.73 \| 1.03 \| 0.69 \| 1.18 \| 0.95 \| 1.06 \| 0.12 \| \| *Fus* \| 1.04 \| \| 0.66 \| 0.99 \| 0.71 \| 0.96 \| 1.07 \| 1.01 \| 0.06 \| \| *Gapdh* \| 1.00 \| \| 1.00 \| 1.00 \| 1.00 \| 1.00 \| 1.00 \| -1.00 \| 0.00 \| \| *Gusb* \| 1.11 \| \| 0.72 \| 1.19 \| 1.00 \| 1.07 \| 1.39 \| 1.23 \| 0.16 \| \| *Hgf* \| ND \| \| ND \| 0.37 \| ND \| ND \| ND \| ND \| ND \| \| *Hif1A* \| 0.91 \| \| 0.73 \| 0.98 \| 0.79 \| 1.08 \| 1.08 \| 1.08 \| 0.00 \| \| *Hnrnpa1* \| 1.01 \| \| 0.67 \| 0.83 \| 0.69 \| 0.83 \| 1.02 \| -1.09 \| 0.09 \| \| *Hnrnph3* \| 1.10 \| \| 1.25 \| 0.55 \| 0.99 \| 0.50 \| 0.79 \| -1.55 \| 0.14 \| \| *Hnrnpk* \| 0.99 \| \| 0.90 \| 0.91 \| 0.88 \| 0.92 \| 0.98 \| -1.06 \| 0.03 \| \| *Hnrnpm* \| 1.02 \| \| 1.03 \| 0.67 \| 0.97 \| 0.66 \| 0.93 \| -1.25 \| 0.14 \| \| *Hnrnpr* \| 1.16 \| \| 0.90 \| 0.69 \| 1.02 \| 0.59 \| 1.13 \| -1.16 \| 0.27 \| \| *Hoxa9* \| 1.08 \| \| 2.35 \| 9.51 \| 1.41 \| 8.83 \| 0.60 \| ND \| ND \| \| *HuR (Elavl1)* \| 0.88 \| \| 0.76 \| 1.06 \| 0.83 \| 1.20 \| 1.09 \| 1.15 \| 0.05 \| \| *Igf1* \| 1.11 \| \| 0.92 \| 0.74 \| 0.69 \| 0.67 \| 0.75 \| -1.41 \| 0.04 \| \| *Igf1R* \| 0.92 \| \| 0.87 \| 0.90 \| 0.68 \| 0.98 \| 0.79 \| -1.13 \| 0.10 \| \| *Igf2R* \| 0.93 \| \| 0.74 \| 1.08 \| 0.88 \| 1.16 \| 1.19 \| 1.17 \| 0.02 \| \| *Irf2* \| 1.18 \| \| 1.05 \| 0.95 \| 0.95 \| 0.80 \| 0.91 \| -1.17 \| 0.05 \| \| *Lamb1* \| 0.74 \| \| 1.03 \| 0.44 \| 0.77 \| 0.60 \| 0.75 \| -1.49 \| 0.07 \| \| *Lef1* \| ND \| \| ND \| 0.41 \| 0.55 \| ND \| ND \| ND \| ND \| \| *Mmp2* \| 1.01 \| \| 0.92 \| 1.84 \| 0.86 \| 1.83 \| 0.94 \| 1.39 \| 0.44 \| \| *Mycbp* \| 0.91 \| \| 0.89 \| 0.69 \| 0.99 \| 0.75 \| 1.11 \| -1.07 \| 0.18 \| \| *Mycl* \| 1.93 \| \| 1.84 \| 2.39 \| 1.52 \| 1.24 \| 0.83 \| 1.03 \| 0.21 \| \| *Ncl* \| 1.12 \| \| 1.10 \| 0.49 \| 0.93 \| 0.44 \| 0.84 \| -1.56 \| 0.20 \| \| *Neat1* \| 0.54 \| \| 0.81 \| 0.43 \| 0.56 \| 0.80 \| 0.68 \| -1.35 \| 0.06 \| \| *Neat1-2* \| 0.61 \| \| 0.50 \| 0.54 \| 0.37 \| 0.89 \| 0.75 \| -1.22 \| 0.07 \| \| *Nfil3* \| 0.80 \| \| 1.08 \| 1.16 \| 0.86 \| 1.45 \| 0.80 \| 1.12 \| 0.33 \| \| *Nkrf (NRF)* \| 0.97 \| \| 1.25 \| 0.84 \| 1.08 \| 0.87 \| 0.86 \| -1.15 \| 0.01 \| \| *Nono* \| 1.21 \| \| 1.26 \| 0.87 \| 0.92 \| 0.72 \| 0.73 \| -1.38 \| 0.01 \| \| *Nr1D1 (rev-erb-a)* \| 1.23 \| \| 1.09 \| 0.86 \| 1.04 \| 0.70 \| 0.95 \| -1.21 \| 0.13 \| \| *Pdgfa* \| 1.12 \| \| 1.03 \| 0.76 \| 1.03 \| 0.68 \| 1.00 \| -1.19 \| 0.16 \| \| *Pdgfb* \| 1.07 \| \| 0.89 \| 1.18 \| 1.07 \| 1.11 \| 1.20 \| 1.15 \| 0.05 \| \| *Per1 (Period-1)* \| 1.18 \| \| 1.13 \| 1.21 \| 1.03 \| 1.03 \| 0.92 \| -1.03 \| 0.05 \| \| *Pfkm (Pfk1)* \| 0.81 \| \| 0.63 \| 0.97 \| 0.57 \| 1.20 \| 0.90 \| 1.05 \| 0.15 \| \| *Pgf (Plgf))* \| 1.02 \| \| 1.00 \| 1.10 \| 1.77 \| 1.09 \| 1.76 \| 1.42 \| 0.34 \| \| *Prox1* \| 0.85 \| \| 0.94 \| 0.88 \| 0.97 \| 1.04 \| 1.03 \| 1.03 \| 0.01 \| \| *Pspc1* \| 1.09 \| \| 1.05 \| 0.65 \| 0.93 \| 0.59 \| 0.89 \| -1.35 \| 0.15 \| \| *Rbm14* \| 1.11 \| \| 0.94 \| 1.03 \| 0.87 \| 0.93 \| 0.93 \| -1.07 \| 0.00 \| \| *Rpl10A* \| 0.91 \| \| 0.82 \| 1.26 \| 1.09 \| 1.39 \| 1.33 \| 1.36 \| 0.03 \| \| *Rps2* \| 1.12 \| \| 0.71 \| 1.23 \| 0.86 \| 1.10 \| 1.22 \| 1.16 \| 0.06 \| \| *Rps25* \| 1.12 \| \| 1.09 \| 0.87 \| 0.99 \| 0.78 \| 0.91 \| -1.19 \| 0.07 \| \| *Rrbp1* \| 0.94 \| \| 0.68 \| 0.58 \| 0.55 \| 0.62 \| 0.82 \| -1.39 \| 0.10 \| \| *Serpine1 (PAI1)* \| 1.03 \| \| 0.62 \| 0.88 \| 0.72 \| 0.86 \| 1.15 \| 1.00 \| 0.15 \| \| *Setd7 (Set7)* \| 1.23 \| \| 0.90 \| 1.47 \| 0.83 \| 1.20 \| 0.92 \| 1.06 \| 0.14 \| \| *Sfpq* \| 0.99 \| \| 1.07 \| 0.71 \| 0.89 \| 0.72 \| 0.83 \| -1.29 \| 0.05 \| \| *Shmt1* \| 1.14 \| \| 0.81 \| 0.98 \| 0.86 \| 0.86 \| 1.06 \| -1.04 \| 0.10 \| \| *Slc7A1 (Cat-1)* \| 1.07 \| \| 0.66 \| 1.06 \| 0.79 \| 0.99 \| 1.21 \| 1.10 \| 0.11 \| \| *Srebf1 (Srebp1)* \| 0.75 \| \| 0.70 \| 0.84 \| 0.78 \| 1.12 \| 1.11 \| 1.12 \| 0.00 \| \| *Sstr2 (Sst2)* \| 1.55 \| \| 1.51 \| 3.86 \| 1.94 \| 2.49 \| 1.28 \| 1.89 \| 0.60 \| \| *Thbd (Thrombomodulin)* \| 1.85 \| \| 0.91 \| 1.05 \| 0.96 \| 0.57 \| 1.06 \| -1.23 \| 0.24 \| \| *Trp53* \| 1.28 \| \| 1.14 \| 1.08 \| 1.09 \| 0.84 \| 0.95 \| -1.11 \| 0.06 \| \| *Txnip* \| 1.13 \| \| 0.74 \| 1.22 \| 0.86 \| 1.07 \| 1.15 \| 1.11 \| 0.04 \| \| *Utrn (Utrophin)* \| 1.04 \| \| 0.83 \| 0.59 \| 0.63 \| 0.57 \| 0.76 \| -1.50 \| 0.09 \| \| *Vash1* \| 1.06 \| \| 0.68 \| 1.13 \| 0.96 \| 1.07 \| 1.41 \| 1.24 \| 0.17 \| \| *Vegfa* \| 0.87 \| \| 0.95 \| 0.83 \| 1.02 \| 0.96 \| 1.07 \| 1.01 \| 0.06 \| \| *Vegfb* \| 0.93 \| \| 0.90 \| 0.75 \| 0.90 \| 0.81 \| 1.00 \| -1.11 \| 0.09 \| \| *Vegfc* \| ND \| \| ND \| 1.16 \| ND \| ND \| ND \| ND \| ND \| \| *Vegfd (Figf)* \| 0.84 \| \| 0.66 \| 0.68 \| 0.99 \| 0.81 \| 1.50 \| 1.15 \| 0.34 \| \| *Xiap* \| 0.97 \| \| 0.79 \| 0.97 \| 0.96 \| 1.00 \| 1.20 \| 1.10 \| 0.10 \| |
| --- | --- | --- | --- | --- | --- | --- | --- | --- | --- | --- | --- | --- | --- | --- | --- | --- | --- | --- | --- | --- | --- | --- | --- | --- | --- | --- | --- | --- | --- | --- | --- | --- | --- | --- | --- | --- | --- | --- | --- | --- | --- | --- | --- | --- | --- | --- | --- | --- | --- | --- | --- | --- | --- | --- | --- | --- | --- | --- | --- | --- | --- | --- | --- | --- | --- | --- | --- | --- | --- | --- | --- | --- | --- | --- | --- | --- | --- | --- | --- | --- | --- | --- | --- | --- | --- | --- | --- | --- | --- | --- | --- | --- | --- | --- | --- | --- | --- | --- | --- | --- | --- | --- | --- | --- | --- | --- | --- | --- | --- | --- | --- | --- | --- | --- | --- | --- | --- | --- | --- | --- | --- | --- | --- | --- | --- | --- | --- | --- | --- | --- | --- | --- | --- | --- | --- | --- | --- | --- | --- | --- | --- | --- | --- | --- | --- | --- | --- | --- | --- | --- | --- | --- | --- | --- | --- | --- | --- | --- | --- | --- | --- | --- | --- | --- | --- | --- | --- | --- | --- | --- | --- | --- | --- | --- | --- | --- | --- | --- | --- | --- | --- | --- | --- | --- | --- | --- | --- | --- | --- | --- | --- | --- | --- | --- | --- | --- | --- | --- | --- | --- | --- | --- | --- | --- | --- | --- | --- | --- | --- | --- | --- | --- | --- | --- | --- | --- | --- | --- | --- | --- | --- | --- | --- | --- | --- | --- | --- | --- | --- | --- | --- | --- | --- | --- | --- | --- | --- | --- | --- | --- | --- | --- | --- | --- | --- | --- | --- | --- | --- | --- | --- | --- | --- | --- | --- | --- | --- | --- | --- | --- | --- | --- | --- | --- | --- | --- | --- | --- | --- | --- | --- | --- | --- | --- | --- | --- | --- | --- | --- | --- | --- | --- | --- | --- | --- | --- | --- | --- | --- | --- | --- | --- | --- | --- | --- | --- | --- | --- | --- | --- | --- | --- | --- | --- | --- | --- | --- | --- | --- | --- | --- | --- | --- | --- | --- | --- | --- | --- | --- | --- | --- | --- | --- | --- | --- | --- | --- | --- | --- | --- | --- | --- | --- | --- | --- | --- | --- | --- | --- | --- | --- | --- | --- | --- | --- | --- | --- | --- | --- | --- | --- | --- | --- | --- | --- | --- | --- | --- | --- | --- | --- | --- | --- | --- | --- | --- | --- | --- | --- | --- | --- | --- | --- | --- | --- | --- | --- | --- | --- | --- | --- | --- | --- | --- | --- | --- | --- | --- | --- | --- | --- | --- | --- | --- | --- | --- | --- | --- | --- | --- | --- | --- | --- | --- | --- | --- | --- | --- | --- | --- | --- | --- | --- | --- | --- | --- | --- | --- | --- | --- | --- | --- | --- | --- | --- | --- | --- | --- | --- | --- | --- | --- | --- | --- | --- | --- | --- | --- | --- | --- | --- | --- | --- | --- | --- | --- | --- | --- | --- | --- | --- | --- | --- | --- | --- | --- | --- | --- | --- | --- | --- | --- | --- | --- | --- | --- | --- | --- | --- | --- | --- | --- | --- | --- | --- | --- | --- | --- | --- | --- | --- | --- | --- | --- | --- | --- | --- | --- | --- | --- | --- | --- | --- | --- | --- | --- | --- | --- | --- | --- | --- | --- | --- | --- | --- | --- | --- | --- | --- | --- | --- | --- | --- | --- | --- | --- | --- | --- | --- | --- | --- | --- | --- | --- | --- | --- | --- | --- | --- | --- | --- | --- | --- | --- | --- | --- | --- | --- | --- | --- | --- | --- | --- | --- | --- | --- | --- | --- | --- | --- | --- | --- | --- | --- | --- | --- | --- | --- | --- | --- | --- | --- | --- | --- | --- | --- | --- | --- | --- | --- | --- | --- | --- | --- | --- | --- | --- | --- | --- | --- | --- | --- | --- | --- | --- | --- | --- | --- | --- | --- | --- | --- | --- | --- | --- | --- | --- | --- | --- | --- | --- | --- | --- | --- | --- | --- | --- | --- | --- | --- | --- | --- | --- | --- | --- | --- | --- | --- | --- | --- | --- | --- | --- | --- | --- | --- | --- | --- | --- | --- | --- | --- | --- | --- | --- | --- | --- | --- | --- | --- | --- | --- | --- | --- | --- | --- | --- | --- | --- | --- | --- | --- | --- | --- | --- | --- | --- | --- | --- | --- | --- | --- | --- | --- | --- | --- | --- | --- | --- | --- | --- | --- | --- | --- | --- | --- | --- | --- | --- | --- | --- | --- | --- | --- | --- | --- | --- | --- | --- | --- | --- | --- | --- | --- | --- | --- | --- | --- | --- | --- | --- | --- | --- | --- | --- | --- | --- | --- | --- | --- | --- | --- | --- | --- | --- | --- | --- | --- | --- | --- | --- | --- | --- | --- | --- | --- | --- | --- | --- | --- | --- | --- | --- | --- | --- | --- | --- | --- | --- | --- | --- | --- | --- | --- | --- | --- | --- | --- | --- | --- | --- | --- | --- | --- | --- | --- | --- | --- | --- | --- | --- | --- | --- | --- | --- | --- | --- | --- | --- | --- | --- | --- | --- | --- | --- | --- | --- | --- | --- | --- | --- | --- | --- | --- | --- | --- | --- | --- | --- | --- | --- | --- | --- | --- | --- | --- | --- | --- | --- | --- | --- | --- | --- | --- | --- | --- | --- | --- | --- | --- | --- | --- | --- | --- | --- | --- | --- | --- | --- | --- | --- | --- | --- | --- | --- | --- | --- | --- | --- | --- | --- | --- | --- | --- | --- | --- | --- | --- | --- | --- | --- | --- | --- | --- | --- | --- | --- | --- | --- | --- | --- | --- | --- | --- | --- | --- | --- | --- | --- | --- | --- | --- | --- | --- | --- | --- | --- | --- | --- | --- | --- | --- | --- | --- | --- | --- | --- | --- | --- | --- | --- | --- | --- | --- | --- | --- | --- | --- | --- | --- | --- | --- | --- | --- | --- | --- | --- | --- | --- | --- | --- | --- | --- | --- | --- | --- | --- | --- | --- | --- | --- | --- | --- | --- | --- | --- | --- | --- | --- | --- | --- | --- | --- | --- | --- | --- | --- | --- | --- | --- | --- | --- | --- | --- | --- | --- | --- | --- | --- | --- | --- | --- | --- | --- | --- | --- | --- | --- | --- | --- | --- | --- | --- | --- | --- | --- | --- | --- | --- | --- | --- | --- | --- | --- | --- | --- | --- | --- | --- | --- | --- | --- | --- | --- | --- | --- | --- | --- | --- | --- | --- | --- | --- | --- | --- | --- | --- | --- | --- | --- | --- | --- | --- | --- | --- | --- | --- | --- | --- | --- |

| **B/ *Neat1_2* knock-down** |  |  |  |  |  |  |  |
| --- | --- | --- | --- | --- | --- | --- | --- |

| \|  \| **Total mRNA** \| \| **Polysome bound mRNA** \| \| **Fold change** \| \| **Fold change mean** \| \| \| --- \| --- \| --- \| --- \| --- \| --- \| --- \| --- \| --- \| \| **Gene name** \| RQ 1 \| RQ 2 \| RQ 1 \| RQ 2 \| RQ polysomes/ RQ Total mRNA 1 \| RQ polysomes/ RQ Total mRNA 2 \| RQ (or -1/RQ) \| SD \| \| *Aanat* \| ND \| 306.15 \| ND \| 0.73 \| ND \| 0.00 \| ND \| ND \| \| *Adar (Adar1)* \| 1.10 \| 1.34 \| 0.88 \| 1.16 \| 0.80 \| 0.87 \| -1.20 \| 0.03 \| \| *Alox15* \| 0.45 \| 2.70 \| 1.98 \| 0.36 \| 4.37 \| 0.13 \| ND \| ND \| \| *Ang* \| 0.83 \| 1.49 \| 0.86 \| 1.09 \| 1.04 \| 0.73 \| -1.13 \| 0.16 \| \| *Apaf1* \| 1.04 \| 1.16 \| 0.93 \| 1.04 \| 0.90 \| 0.90 \| -1.11 \| 0.00 \| \| *Apln* \| 1.23 \| 1.53 \| 0.76 \| 1.54 \| 0.62 \| 1.00 \| -1.23 \| 0.19 \| \| *Aplnr* \| ND \| 3.03 \| 0.97 \| 1.74 \| ND \| 0.57 \| ND \| ND \| \| *Bag1* \| 1.28 \| 1.38 \| 1.27 \| 1.04 \| 0.99 \| 0.76 \| -1.15 \| 0.12 \| \| *Bax* \| 1.15 \| 1.00 \| 0.79 \| 1.00 \| 0.69 \| 1.00 \| -1.18 \| 0.16 \| \| *Bcl2 (Bcl-2)* \| 1.04 \| 1.38 \| 0.91 \| 1.16 \| 0.88 \| 0.84 \| -1.16 \| 0.02 \| \| *Bcl2L1 (Bcl-XL)* \| 1.19 \| 1.20 \| 0.96 \| 1.12 \| 0.81 \| 0.93 \| -1.15 \| 0.06 \| \| *Bip (GRP78/Hspa5)* \| 0.78 \| 1.16 \| 0.67 \| 0.92 \| 0.87 \| 0.79 \| -1.21 \| 0.04 \| \| *BNP (Nppb)* \| 0.83 \| 0.60 \| 0.61 \| 0.88 \| 0.74 \| 1.48 \| 1.11 \| 0.37 \| \| *Casp2 (Caspase-2)* \| 1.02 \| 1.30 \| 0.99 \| 0.99 \| 0.97 \| 0.76 \| -1.15 \| 0.10 \| \| *Cdk1* \| 1.10 \| 1.19 \| 1.00 \| 1.08 \| 0.91 \| 0.91 \| -1.10 \| 0.00 \| \| *Col1A1* \| 1.52 \| 1.35 \| 0.76 \| 1.00 \| 0.50 \| 0.74 \| -1.61 \| 0.12 \| \| *Col3A1* \| 1.47 \| 1.08 \| 0.38 \| 1.05 \| 0.25 \| 0.98 \| -1.62 \| 0.36 \| \| *Csde1* \| 1.21 \| 1.48 \| 1.18 \| 0.99 \| 0.98 \| 0.67 \| -1.22 \| 0.15 \| \| *Ctgf* \| 1.09 \| 1.47 \| 0.74 \| 0.99 \| 0.68 \| 0.67 \| -1.48 \| 0.00 \| \| *Cyr61* \| 1.08 \| 1.00 \| 0.73 \| 1.02 \| 0.68 \| 1.03 \| -1.17 \| 0.17 \| \| *Dazap1* \| 1.15 \| 1.35 \| 1.05 \| 0.99 \| 0.91 \| 0.73 \| -1.22 \| 0.09 \| \| *Ddx17* \| 1.12 \| 1.22 \| 0.85 \| 1.17 \| 0.76 \| 0.95 \| -1.17 \| 0.10 \| \| *Egr1* \| 0.96 \| 1.16 \| 0.80 \| 0.90 \| 0.83 \| 0.78 \| -1.24 \| 0.03 \| \| *Eno3* \| 1.03 \| 1.36 \| 0.93 \| 1.08 \| 0.90 \| 0.80 \| -1.18 \| 0.05 \| \| *Epas1* \| 1.17 \| 1.58 \| 0.95 \| 1.02 \| 0.82 \| 0.64 \| -1.37 \| 0.09 \| \| *Fgf1* \| 1.09 \| 1.45 \| 1.00 \| 0.95 \| 0.92 \| 0.65 \| -1.27 \| 0.13 \| \| *Fgf10* \| 0.90 \| 1.26 \| 0.25 \| 2.94 \| 0.27 \| 2.33 \| ND \| ND \| \| *Fgf2* \| 4.34 \| 2.07 \| 0.85 \| 2.41 \| 0.20 \| 1.16 \| ND \| ND \| \| *Fgf21* \| 0.82 \| 0.97 \| 0.67 \| 1.01 \| 0.82 \| 1.03 \| -1.08 \| 0.10 \| \| *Fgf23* \| ND \| ND \| ND \| 0.86 \| ND \| ND \| ND \| ND \| \| *Fgf4* \| 10.86 \| 1.25 \| 1.93 \| 1.48 \| 0.18 \| 1.18 \| -1.47 \| 0.50 \| \| *Fgfr1* \| 1.19 \| 1.86 \| 0.66 \| 1.04 \| 0.56 \| 0.56 \| -1.79 \| 0.00 \| \| *Fgfr2* \| 0.70 \| 0.79 \| 1.13 \| 1.44 \| 1.61 \| 1.83 \| 1.72 \| 0.11 \| \| *Fgfr3* \| 0.96 \| 1.39 \| 0.77 \| 1.06 \| 0.80 \| 0.77 \| -1.28 \| 0.02 \| \| *Fmr1 (Fmrp)* \| 1.30 \| 1.67 \| 1.17 \| 1.31 \| 0.90 \| 0.78 \| -1.19 \| 0.06 \| \| *Fn1 (Fibronectine)* \| 0.96 \| 1.41 \| 0.86 \| 1.46 \| 0.89 \| 1.04 \| -1.04 \| 0.07 \| \| *Fus* \| 0.99 \| 1.26 \| 0.89 \| 1.09 \| 0.90 \| 0.86 \| -1.14 \| 0.02 \| \| *Gapdh* \| 1.00 \| 1.00 \| 1.00 \| 1.00 \| 1.00 \| 1.00 \| -1.00 \| 0.00 \| \| *Gusb* \| 1.09 \| 1.26 \| 0.88 \| 1.13 \| 0.81 \| 0.89 \| -1.17 \| 0.04 \| \| *Hgf* \| ND \| ND \| ND \| ND \| ND \| ND \| ND \| ND \| \| *Hif1A* \| 0.99 \| 1.18 \| 1.01 \| 1.03 \| 1.02 \| 0.87 \| -1.06 \| 0.08 \| \| *Hnrnpa1* \| 1.10 \| 1.14 \| 0.96 \| 1.07 \| 0.87 \| 0.94 \| -1.10 \| 0.04 \| \| *Hnrnph3* \| 1.01 \| 1.59 \| 0.85 \| 1.14 \| 0.85 \| 0.72 \| -1.28 \| 0.06 \| \| *Hnrnpk* \| 1.11 \| 1.03 \| 0.94 \| 1.06 \| 0.85 \| 1.02 \| -1.07 \| 0.09 \| \| *Hnrnpm* \| 1.04 \| 1.50 \| 1.02 \| 1.21 \| 0.98 \| 0.81 \| -1.12 \| 0.08 \| \| *Hnrnpr* \| 1.22 \| 1.44 \| 1.11 \| 1.16 \| 0.91 \| 0.81 \| -1.16 \| 0.05 \| \| *Hoxa9* \| 0.65 \| 0.60 \| 0.52 \| 0.19 \| 0.80 \| 0.31 \| -1.80 \| 0.24 \| \| *HuR (Elavl1)* \| 1.04 \| 1.35 \| 0.97 \| 0.99 \| 0.93 \| 0.73 \| -1.20 \| 0.10 \| \| *Igf1* \| 1.98 \| 2.38 \| 0.54 \| 1.10 \| 0.27 \| 0.46 \| -2.72 \| 0.10 \| \| *Igf1R* \| 0.83 \| 1.28 \| 0.67 \| 0.97 \| 0.81 \| 0.76 \| -1.27 \| 0.02 \| \| *Igf2R* \| 0.88 \| 1.43 \| 0.88 \| 1.11 \| 1.00 \| 0.78 \| -1.13 \| 0.11 \| \| *Irf2* \| 1.20 \| 1.21 \| 1.08 \| 1.04 \| 0.90 \| 0.86 \| -1.14 \| 0.02 \| \| *Lamb1* \| 1.22 \| 1.58 \| 1.25 \| 1.23 \| 1.03 \| 0.77 \| -1.11 \| 0.13 \| \| *Lef1* \| ND \| ND \| 2.27 \| 2.18 \| ND \| ND \| ND \| ND \| \| *Mmp2* \| 0.84 \| 2.21 \| 0.81 \| 0.97 \| 0.96 \| 0.44 \| -1.42 \| 0.26 \| \| *Mycbp* \| 1.33 \| 1.22 \| 0.87 \| 1.03 \| 0.65 \| 0.85 \| -1.33 \| 0.10 \| \| *Mycl* \| 1.48 \| 1.02 \| 0.92 \| 0.95 \| 0.62 \| 0.93 \| -1.29 \| 0.16 \| \| *Ncl* \| 1.18 \| 1.63 \| 0.99 \| 1.01 \| 0.84 \| 0.62 \| -1.37 \| 0.11 \| \| *Neat1* \| 0.96 \| 1.30 \| 0.54 \| 0.87 \| 0.57 \| 0.67 \| -1.62 \| 0.05 \| \| *Neat1-2* \| 0.37 \| 0.78 \| 0.41 \| 0.59 \| 1.09 \| 0.75 \| -1.08 \| 0.17 \| \| *Nfil3* \| 1.47 \| 1.35 \| 1.28 \| 1.10 \| 0.87 \| 0.82 \| -1.18 \| 0.03 \| \| *Nkrf (NRF)* \| 1.32 \| 1.85 \| 1.13 \| 1.14 \| 0.85 \| 0.62 \| -1.36 \| 0.12 \| \| *Nono* \| 1.27 \| 1.49 \| 1.06 \| 1.21 \| 0.84 \| 0.81 \| -1.22 \| 0.01 \| \| *Nr1D1 (rev-erb-a)* \| 1.04 \| 1.18 \| 0.87 \| 1.03 \| 0.83 \| 0.87 \| -1.17 \| 0.02 \| \| *p16INK4 (Cdkn2A)* \| 1.07 \| 0.98 \| 0.83 \| 1.31 \| 0.78 \| 1.33 \| 1.06 \| 0.28 \| \| *p27kip1 (Cdkn1B)* \| 1.17 \| 1.39 \| 0.99 \| 1.06 \| 0.85 \| 0.77 \| -1.24 \| 0.04 \| \| *Pdgfa* \| 1.06 \| 1.32 \| 0.85 \| 1.15 \| 0.80 \| 0.87 \| -1.20 \| 0.04 \| \| *Pdgfb* \| 1.46 \| 1.16 \| 0.86 \| 1.20 \| 0.59 \| 1.03 \| -1.23 \| 0.22 \| \| *Per1 (Period-1)* \| 1.17 \| 1.49 \| 0.99 \| 1.04 \| 0.84 \| 0.70 \| -1.30 \| 0.07 \| \| *Pfkm (Pfk1)* \| 0.96 \| 1.18 \| 0.89 \| 1.02 \| 0.92 \| 0.86 \| -1.12 \| 0.03 \| \| *Pgf (Plgf)* \| 1.19 \| 1.09 \| 1.32 \| 1.43 \| 1.11 \| 1.31 \| -0.83 \| 0.10 \| \| *Prox1* \| 1.26 \| 1.68 \| 1.23 \| 1.25 \| 0.98 \| 0.74 \| -1.16 \| 0.12 \| \| *Pspc1* \| 1.07 \| 1.17 \| 0.97 \| 1.13 \| 0.91 \| 0.97 \| -1.07 \| 0.03 \| \| *Rbm14* \| 1.12 \| 1.44 \| 0.91 \| 1.11 \| 0.82 \| 0.77 \| -1.26 \| 0.02 \| \| *Rpl10A* \| 0.91 \| 0.88 \| 0.98 \| 1.04 \| 1.07 \| 1.18 \| 1.12 \| 0.06 \| \| *Rps2* \| 0.94 \| 1.14 \| 0.86 \| 0.96 \| 0.92 \| 0.84 \| -1.14 \| 0.04 \| \| *Rps25* \| 0.69 \| 1.10 \| 0.93 \| 0.88 \| 1.34 \| 0.80 \| 1.07 \| 0.27 \| \| *Rrbp1* \| 0.90 \| 1.11 \| 0.65 \| 1.13 \| 0.72 \| 1.02 \| -1.15 \| 0.15 \| \| *Serpine1 (PAI1)* \| 0.74 \| 1.51 \| 0.85 \| 1.07 \| 1.15 \| 0.71 \| -1.08 \| 0.22 \| \| *Setd7 (Set7)* \| 1.19 \| 1.36 \| 1.02 \| 0.99 \| 0.86 \| 0.73 \| -1.26 \| 0.06 \| \| *Sfpq* \| 1.13 \| 1.90 \| 1.00 \| 1.30 \| 0.88 \| 0.69 \| -1.27 \| 0.10 \| \| *Shmt1* \| 1.14 \| 1.39 \| 0.99 \| 1.19 \| 0.87 \| 0.86 \| -1.15 \| 0.00 \| \| *Slc7A1 (Cat-1)* \| 0.87 \| 1.49 \| 0.74 \| 1.02 \| 0.85 \| 0.68 \| -1.30 \| 0.08 \| \| *Srebf1 (Srebp1)* \| 1.00 \| 1.59 \| 0.64 \| 1.11 \| 0.64 \| 0.70 \| -1.49 \| 0.03 \| \| *Sstr2 (Sst2)* \| 0.65 \| 0.98 \| 0.87 \| 1.12 \| 1.34 \| 1.14 \| 1.24 \| 0.10 \| \| *Thbd (Thrombomodulin)* \| 1.05 \| 0.74 \| 0.51 \| 0.51 \| 0.48 \| 0.69 \| -1.71 \| 0.10 \| \| *Trp53* \| 1.14 \| 1.25 \| 0.95 \| 0.98 \| 0.83 \| 0.79 \| -1.24 \| 0.02 \| \| *Txnip* \| 1.17 \| 1.29 \| 1.02 \| 0.99 \| 0.87 \| 0.77 \| -1.22 \| 0.05 \| \| *Utrn (Utrophin)* \| 0.93 \| 1.59 \| 0.99 \| 1.10 \| 1.07 \| 0.69 \| -1.14 \| 0.19 \| \| *Vash1* \| 1.41 \| 1.82 \| 1.05 \| 1.02 \| 0.74 \| 0.56 \| -1.54 \| 0.09 \| \| *Vegfa* \| 1.02 \| 1.64 \| 0.88 \| 1.01 \| 0.86 \| 0.62 \| -1.35 \| 0.12 \| \| *Vegfb* \| 1.40 \| 1.58 \| 1.02 \| 1.06 \| 0.73 \| 0.67 \| -1.43 \| 0.03 \| \| *Vegfc* \| ND \| ND \| 1.20 \| ND \| ND \| ND \| ND \| ND \| \| *Vegfd (Figf)* \| 1.23 \| 1.13 \| 1.06 \| 1.04 \| 0.86 \| 0.93 \| -1.12 \| 0.03 \| \| *Xiap* \| 1.44 \| 1.45 \| 1.07 \| 1.10 \| 0.74 \| 0.76 \| -1.33 \| 0.01 \| |
| --- | --- | --- | --- | --- | --- | --- | --- | --- | --- | --- | --- | --- | --- | --- | --- | --- | --- | --- | --- | --- | --- | --- | --- | --- | --- | --- | --- | --- | --- | --- | --- | --- | --- | --- | --- | --- | --- | --- | --- | --- | --- | --- | --- | --- | --- | --- | --- | --- | --- | --- | --- | --- | --- | --- | --- | --- | --- | --- | --- | --- | --- | --- | --- | --- | --- | --- | --- | --- | --- | --- | --- | --- | --- | --- | --- | --- | --- | --- | --- | --- | --- | --- | --- | --- | --- | --- | --- | --- | --- | --- | --- | --- | --- | --- | --- | --- | --- | --- | --- | --- | --- | --- | --- | --- | --- | --- | --- | --- | --- | --- | --- | --- | --- | --- | --- | --- | --- | --- | --- | --- | --- | --- | --- | --- | --- | --- | --- | --- | --- | --- | --- | --- | --- | --- | --- | --- | --- | --- | --- | --- | --- | --- | --- | --- | --- | --- | --- | --- | --- | --- | --- | --- | --- | --- | --- | --- | --- | --- | --- | --- | --- | --- | --- | --- | --- | --- | --- | --- | --- | --- | --- | --- | --- | --- | --- | --- | --- | --- | --- | --- | --- | --- | --- | --- | --- | --- | --- | --- | --- | --- | --- | --- | --- | --- | --- | --- | --- | --- | --- | --- | --- | --- | --- | --- | --- | --- | --- | --- | --- | --- | --- | --- | --- | --- | --- | --- | --- | --- | --- | --- | --- | --- | --- | --- | --- | --- | --- | --- | --- | --- | --- | --- | --- | --- | --- | --- | --- | --- | --- | --- | --- | --- | --- | --- | --- | --- | --- | --- | --- | --- | --- | --- | --- | --- | --- | --- | --- | --- | --- | --- | --- | --- | --- | --- | --- | --- | --- | --- | --- | --- | --- | --- | --- | --- | --- | --- | --- | --- | --- | --- | --- | --- | --- | --- | --- | --- | --- | --- | --- | --- | --- | --- | --- | --- | --- | --- | --- | --- | --- | --- | --- | --- | --- | --- | --- | --- | --- | --- | --- | --- | --- | --- | --- | --- | --- | --- | --- | --- | --- | --- | --- | --- | --- | --- | --- | --- | --- | --- | --- | --- | --- | --- | --- | --- | --- | --- | --- | --- | --- | --- | --- | --- | --- | --- | --- | --- | --- | --- | --- | --- | --- | --- | --- | --- | --- | --- | --- | --- | --- | --- | --- | --- | --- | --- | --- | --- | --- | --- | --- | --- | --- | --- | --- | --- | --- | --- | --- | --- | --- | --- | --- | --- | --- | --- | --- | --- | --- | --- | --- | --- | --- | --- | --- | --- | --- | --- | --- | --- | --- | --- | --- | --- | --- | --- | --- | --- | --- | --- | --- | --- | --- | --- | --- | --- | --- | --- | --- | --- | --- | --- | --- | --- | --- | --- | --- | --- | --- | --- | --- | --- | --- | --- | --- | --- | --- | --- | --- | --- | --- | --- | --- | --- | --- | --- | --- | --- | --- | --- | --- | --- | --- | --- | --- | --- | --- | --- | --- | --- | --- | --- | --- | --- | --- | --- | --- | --- | --- | --- | --- | --- | --- | --- | --- | --- | --- | --- | --- | --- | --- | --- | --- | --- | --- | --- | --- | --- | --- | --- | --- | --- | --- | --- | --- | --- | --- | --- | --- | --- | --- | --- | --- | --- | --- | --- | --- | --- | --- | --- | --- | --- | --- | --- | --- | --- | --- | --- | --- | --- | --- | --- | --- | --- | --- | --- | --- | --- | --- | --- | --- | --- | --- | --- | --- | --- | --- | --- | --- | --- | --- | --- | --- | --- | --- | --- | --- | --- | --- | --- | --- | --- | --- | --- | --- | --- | --- | --- | --- | --- | --- | --- | --- | --- | --- | --- | --- | --- | --- | --- | --- | --- | --- | --- | --- | --- | --- | --- | --- | --- | --- | --- | --- | --- | --- | --- | --- | --- | --- | --- | --- | --- | --- | --- | --- | --- | --- | --- | --- | --- | --- | --- | --- | --- | --- | --- | --- | --- | --- | --- | --- | --- | --- | --- | --- | --- | --- | --- | --- | --- | --- | --- | --- | --- | --- | --- | --- | --- | --- | --- | --- | --- | --- | --- | --- | --- | --- | --- | --- | --- | --- | --- | --- | --- | --- | --- | --- | --- | --- | --- | --- | --- | --- | --- | --- | --- | --- | --- | --- | --- | --- | --- | --- | --- | --- | --- | --- | --- | --- | --- | --- | --- | --- | --- | --- | --- | --- | --- | --- | --- | --- | --- | --- | --- | --- | --- | --- | --- | --- | --- | --- | --- | --- | --- | --- | --- | --- | --- | --- | --- | --- | --- | --- | --- | --- | --- | --- | --- | --- | --- | --- | --- | --- | --- | --- | --- | --- | --- | --- | --- | --- | --- | --- | --- | --- | --- | --- | --- | --- | --- | --- | --- | --- | --- | --- | --- | --- | --- | --- | --- | --- | --- | --- | --- | --- | --- | --- | --- | --- | --- | --- | --- | --- | --- | --- | --- | --- | --- | --- | --- | --- | --- | --- | --- | --- | --- | --- | --- | --- | --- | --- | --- | --- | --- | --- | --- | --- | --- | --- | --- | --- | --- | --- | --- | --- | --- | --- | --- | --- | --- | --- | --- | --- | --- | --- | --- | --- | --- | --- | --- | --- | --- | --- | --- | --- | --- | --- | --- | --- | --- | --- | --- | --- | --- | --- | --- | --- | --- | --- | --- | --- | --- | --- | --- | --- | --- | --- | --- | --- | --- | --- | --- | --- | --- | --- | --- | --- | --- | --- | --- | --- | --- | --- | --- | --- | --- | --- | --- | --- | --- | --- | --- | --- | --- | --- | --- | --- | --- | --- | --- | --- | --- | --- | --- | --- | --- | --- | --- | --- | --- | --- | --- | --- | --- | --- |
